# COMER2: GPU-accelerated sensitive and specific homology searches

**DOI:** 10.1093/bioinformatics/btaa185

**Published:** 2020-03-13

**Authors:** Mindaugas Margelevičius

**Affiliations:** Institute of Biotechnology, Life Sciences Center, Vilnius University, Vilnius 10257, Lithuania

## Abstract

**Summary:**

Searching for homology in the vast amount of sequence data has a particular emphasis on its speed. We present a completely rewritten version of the sensitive homology search method COMER based on alignment of protein sequence profiles, which is capable of searching big databases even on a lightweight laptop. By harnessing the power of CUDA-enabled graphics processing units, it is up to 20 times faster than HHsearch, a state-of-the-art method using vectorized instructions on modern CPUs.

**Availability and implementation:**

COMER2 is cross-platform open-source software available at https://sourceforge.net/projects/comer2 and https://github.com/minmarg/comer2. It can be easily installed from source code or using stand-alone installers.

**Contact:**

mindaugas.margelevicius@bti.vu.lt

**Supplementary information:**

Supplementary data are available at *Bioinformatics* online.

## 1 Introduction

The detection of homology between protein sequences underlies a wide variety of protein studies ranging from active site and function prediction to contact and structure prediction. Sensitivity, therefore, is one of the most desirable properties of homology search methods. Efforts to increase sensitivity, however, are often accompanied by an increase in computation time. For homology search methods to be applicable to the vast amount of sequence data that is available today, various algorithms have been proposed to reduce computation time (Altschul *et al.*, 1997; Eddy, 2011; Raimondi *et al.*, 2018; Remmert *et al.*, 2012).

This article contributes to increasing computing performance and presents a new implementation (version 2.2) of the COMER method (Margelevičius, 2016, 2018) for homology search based on alignment of protein sequence profiles. The new version is more than three orders of magnitude faster, more sensitive, produces alignments of higher quality and, as it is shown, highly specific.

## 2 Materials and methods

COMER computing performance is increased by harnessing the power of the graphics processing unit (GPU). To the best of our knowledge, COMER2 represents the first GPU implementation of homology searches based on profile–profile alignment. As a target processor, a GPU was selected because of its high level of parallelism and cost efficiency.

The approach and algorithms behind the COMER method have not changed, but the software has been completely rewritten (see Supplementary Section S1). Changed architecture, data types, fast approximations to exponential/logarithmic functions and other code optimizations have an impact on sensitivity and alignment quality, which we evaluate in addition to computation time.

We evaluated and benchmarked the new software on (i) a laptop equipped with a six-core hyper-threaded Intel Core i9-8950HK CPU clocked at 2.9 GHz, 16GB DDR4 RAM and an 8GB GDDR5 NVIDIA GeForce GTX 1080MQ GPU and (ii) a high-end server with two 10-core hyper-threaded Intel Xeon Gold 5115 CPUs at 2.4 GHz, 128GB DDR4 RAM and two 16GB HBM2 NVIDIA Tesla V100 GPU accelerators. The CPUs of these computers support the AVX2/AVX512 instruction set that provides maximum instruction throughput for HHsearch/HHblits v3.2.

Two datasets were used. The first, referred to as the SCOPe dataset, was half of the entries in the SCOPe 2.03 database (Fox *et al.*, 2013) filtered at 20% sequence identity. Profiles were constructed from multiple sequence alignments (MSAs) obtained using (i) PSI-BLAST (Altschul *et al.*, 1997) and (ii) HHblits (Remmert *et al.*, 2012). 1722 profiles were used as queries to search a total of 4900 profiles.

The second, referred to as the simulated dataset, was composed of 1 million randomly generated profiles (Margelevičius, 2019) and 1284 profiles constructed for the sequences of randomly selected PDB (Berman *et al.*, 2000) structures sharing <20% sequence identity at the domain level (1 001 284 profiles in total). Another set of 128 profiles corresponding to PDB entries was used as queries. MSAs for the PDB sequences were obtained from HHpred PDB70 database 190918 (Zimmermann *et al.*, 2018).

Sensitivity was measured by the number of identified true positives (TPs). A TP was a domain that was structurally similar to the query domain or belonged to the same SCOPe superfamily as the query. For both datasets, the analysis was conducted at the domain level. A hit to a dissimilar domain from a different fold or to a random profile was a false positive (FP).

For the SCOPe dataset, alignment quality was evaluated by generating structural models based on profile–profile alignments. A high-quality alignment (HQA) corresponded to a model structurally similar to the native structure. Alignment quality was evaluated locally, along the alignment extent and globally, regarding the whole domain.

More details can be found in Supplementary Section S2.

## 3 Results

**Fig. 1.**
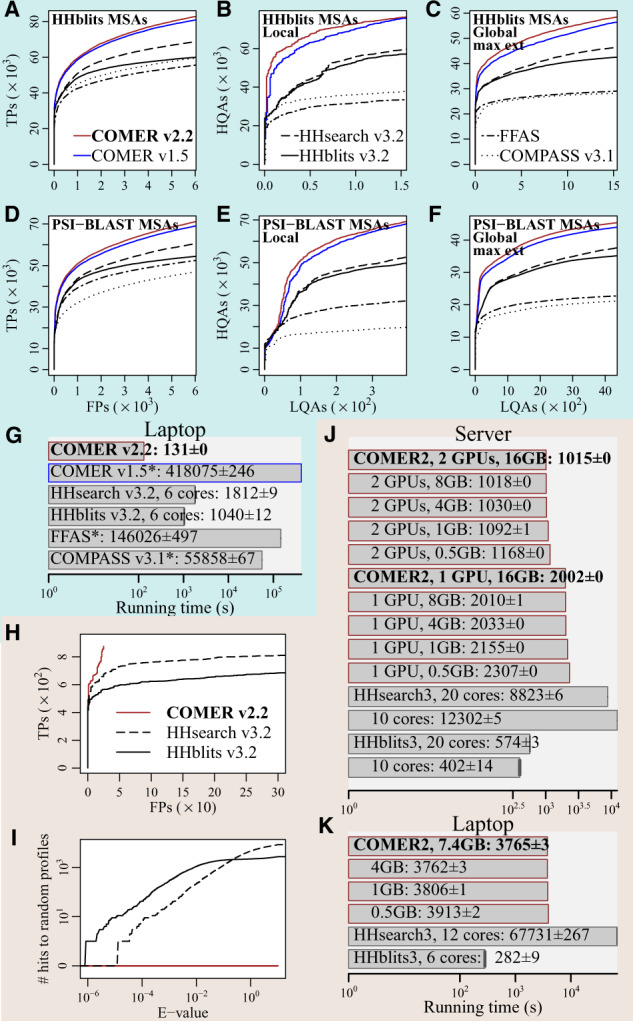
Results for (**A–G**) the SCOPe and (**H–K**) the simulated dataset. (A, D and H) sensitivity; (B and E) alignment quality in the local mode; (C and F) alignment quality in the global mode. COMER, HHsearch and HHblits maximally extend alignments. (G, J and K) running time; (I) specificity. Bold type denotes the new COMER version. *Measured on a cluster with Intel Xeon CPUs E5-2670 v3

The results accumulated over all queries are shown in Figure 1. More results can be found in Supplementary Section S3.

The new version for the SCOPe dataset is more sensitive (Fig. 1A and D), produces higher-quality alignments evaluated locally (Fig. 1B and E) and globally (Fig. 1C and F) and is more than three orders of magnitude faster (Fig. 1G) when measured on the laptop.

The simulated dataset (53GB) was used to estimate running time on big data and evaluate specificity. COMER v2.2 and HHsearch/HHblits v3.2 (Steinegger *et al.*, 2019) were the only feasible implementations for this test.

The results show that COMER v2.2 is highly sensitive (Fig. 1H) and specific (Fig. 1I). All FPs but one were associated with the same query (1AL3_A) and locally shared the same secondary structure with it, indicating a possible evolutionary signal. And there were no hits to random profiles. In contrast, the results of HHsearch and HHblits included many statistically significant alignments between the queries and random profiles.

The property of simultaneously performing searches with multiple queries and the capability of utilizing multiple GPUs help COMER v2.2 achieve high performance and scalability. COMER v2.2 is nearly 10× and 20× faster than HHsearch v3.2, measured on the server (Fig. 1J) and the laptop (Fig. 1K), respectively. A beneficial characteristic is a weak dependence of its running time (varies within 15%) on the GPU memory used.

The gains in running time achieved by HHblits are due to its filtering strategy that identified only 7870 (0.8%) profile pairs per query on average as candidates for alignment, whereas this number for COMER corresponds to the total number of entries in the database. This difference in computational complexity indicates a high potential to significantly increase COMER’s computing performance further (see Section 4).

## 4 Discussion

COMER v2.2 is cross-platform software written in CUDA and C++11 and designed to run on major operating systems (Windows, Linux and macOS). It was tested on Windows 10 using the native compiler and on the Ubuntu 18.04 LTS and CentOS 7.6 Linux distributions using GCC (4.8.5, 7.4 and 8.3) and LLVM/Clang 6.0 compilers.

The new implementation of the COMER method exhibits good performance characteristics and advantageous features. The GPU memory limit that can be used by COMER2 is configurable, and the performance weakly depends on the amount of memory used for computation. In other words, the efficient use of computational resources allows for fast searching big databases even on a lightweight laptop.

COMER2 is capable of utilizing multiple GPUs and demonstrates excellent scalability, which enables large-scale sensitive homology search to be performed within minutes. The performance gain is achieved without compromising sensitivity and alignment quality. On the contrary, COMER2 is now more sensitive and produces more accurate alignments.

The software architecture provides a foundation for further significant improvements. The current version calculates complete dynamic programming (DP) matrices and does not use any heuristics to screen for candidates to the profile–profile alignment phase. Implementing algorithms for reducing DP complexity, such as extending from seeds found in the score matrix and applying banded DP, and for filtering out possibly unrelated pairs can lead to orders of magnitude improvement in performance. We will investigate these and other directions in future work.

## Supplementary Material

btaa185_Supplementary_Data

## References

[btaa185-B1] Altschul S.F. et al (1997) Gapped BLAST and PSI-BLAST: a new generation of protein database search programs. Nucleic Acids Res., 25, 3389–3402. 9254694 10.1093/nar/25.17.3389 PMC146917

[btaa185-B2] Berman H. (2000) The Protein Data Bank. Nucleic Acids Res., 28, 235–242. 10592235 10.1093/nar/28.1.235 PMC102472

[btaa185-B3] Eddy S. (2011) Accelerated profile HMM searches. PLoS Comput. Biol., 7, e1002195. 22039361 10.1371/journal.pcbi.1002195 PMC3197634

[btaa185-B4] Fox N. et al (2014) SCOPe: Structural classification of proteins–extended, integrating SCOP and ASTRAL data and classification of new structures. Nucleic Acids Res., 42, D304–D309. 24304899 10.1093/nar/gkt1240 PMC3965108

[btaa185-B5] Margelevičius M. (2016) Bayesian nonparametrics in protein remote homology search. Bioinformatics, 32, 2744–2752. 27153649 10.1093/bioinformatics/btw213

[btaa185-B6] Margelevičius M. (2018) A low-complexity add-on score for protein remote homology search with COMER. Bioinformatics, 34, 2037–2045. 29390109 10.1093/bioinformatics/bty048

[btaa185-B7] Margelevičius M. (2019) Estimating statistical significance of local protein profile-profile alignments. BMC Bioinformatics, 20, 419. 31409275 10.1186/s12859-019-2913-3 PMC6693267

[btaa185-B8] Raimondi D. et al (2018) Ultra-fast global homology detection with Discrete Cosine Transform and Dynamic Time Warping. Bioinformatics, 34, 3118–3125. 29684140 10.1093/bioinformatics/bty309

[btaa185-B9] Remmert M. et al (2012) HHblits: lightning-fast iterative protein sequence searching by HMM-HMM alignment. Nat. Methods, 9, 173–175. 10.1038/nmeth.1818 22198341

[btaa185-B10] Steinegger M. et al (2019) HH-suite3 for fast remote homology detection and deep protein annotation. BMC Bioinformatics, 20, 473. 31521110 10.1186/s12859-019-3019-7 PMC6744700

[btaa185-B11] Zimmermann L. et al (2018) A completely reimplemented MPI bioinformatics toolkit with a new HHpred server at its core. J. Mol. Biol., 430, 2237–2243. 29258817 10.1016/j.jmb.2017.12.007

