# Supplementary material for: COMER2: GPU-accelerated sensitive and specific homology searches
Source: Bioinformatics. 2020 Mar 13;36(11):3570–2. doi: 10.1093/bioinformatics/btaa185 (PMC7267824; doi:10.1093/bioinformatics/btaa185)
Supplement: btaa185_Supplementary_Data [file btaa185_supplementary_data.pdf]

COMER2: GPU-accelerated sensitive and specific homology searches

Supplementary Materials

Mindaugas Margelevičius

Institute of Biotechnology, Life Sciences Center, Vilnius University, Vilnius, Lithuania

mindaugas.margelevicius@bti.vu.lt

Contents

S1 Methods **2**
S1.1 COMER2 process . . . 2
S1.2 Data representation in memory . . . 3
S1.3 Computation of scores . . . 3
S1.4 Computation of DP matrices . . . 4
S1.5 Impact on sensitivity and alignment quality . . . 5

S2 Evaluation **7**
S2.1 Datasets . . . 7
S2.1.1 SCOPe dataset . . . 7
S2.1.2 Simulated dataset . . . 7
S2.2 Command-line options of profile-profile alignment methods . . . 8
S2.3 Running time . . . 9
S2.4 Sensitivity . . . 9
S2.5 Alignment quality . . . 9

S3 Results **9**
S3.1 Additional results for the SCOPe dataset . . . 9
S3.2 Running time for the simulated dataset . . . 12
S3.3 Benchmark tests on multi-domain proteins . . . 13
S3.4 Running time of profile and database construction . . . 16

S4 Software and data availability **17**

References **18**

S1 Methods

This section provides an overview of algorithms implemented in COMER v2.2 for accelerating profile-profile comparison and alignment on the GPU.

S1.1 COMER2 process

The schematic diagram of COMER2 process using multiple GPUs is shown in Figure S1. To utilize the thousand-core architecture of the GPU, data (profiles) are processed in large chunks read constantly by the *Data reader* thread. The size of data chunks depends on the GPU memory allocated for computation and the length of the longest query profile. The *Job dispatcher* thread on the CPU iteratively instructs the *Commutator* threads to distribute data chunks across one or more GPUs and initiate computation.

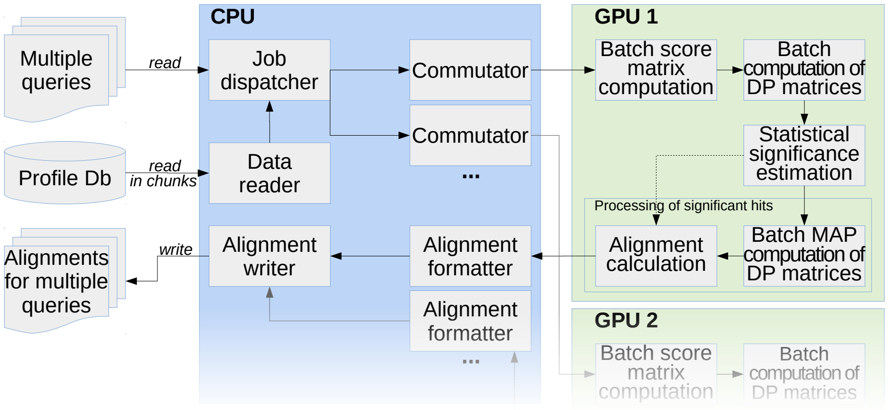

Figure S1. **Schematic diagram of COMER2 process.**

A GPU performs complete alignment computation on an assigned chunk of data for each query profile in parallel. First, a batch score matrix between the query profiles and the multiple database profiles of the data chunk is constructed. The batch score matrix is used to batch calculate dynamic programming (DP) matrices. A challenging aspect is parallelization of DP, which introduces dependencies between the cells of the matrix. Next, calculated alignment scores and estimated statistical significance of the alignments (Margelevičius, 2019) determine which database profiles progress to the next phase. Optionally, DP matrices for selected profiles are recalculated using a maximum a posteriori (MAP) DP algorithm (Durbin *et al.*, 1998, p. 94). Final alignments are obtained by backtracking through the (MAP) DP matrices. The alignments computed on the GPU are further transferred to an *Alignment formatter* thread on the CPU, which formats them for output and passes the data to the *Alignment writer* responsible for merging and writing the final alignments to disk once all database profiles have been processed.

The following subsections focus on the aspects affecting COMER2 computing performance the most. That is data representation, batch computation of a score matrix, and batch computation of DP matrices, which is common to both DP and MAP DP matrices.

S1.2 Data representation in memory

Organization of data in memory is highly important when targeting performance. The highest memory throughput can be achieved when adjacent memory locations are accessed for data. Therefore, profiles are organized in memory as a collection of large continuous arrays of attribute values, which represent amino acid distribution, transition probabilities, context and secondary structure data, and others (Figure S2). One array (cells along the horizontal axis) represents one attribute (e.g., probability of a certain amino acid). And each array is physically independent and can map to a disjoint memory region.

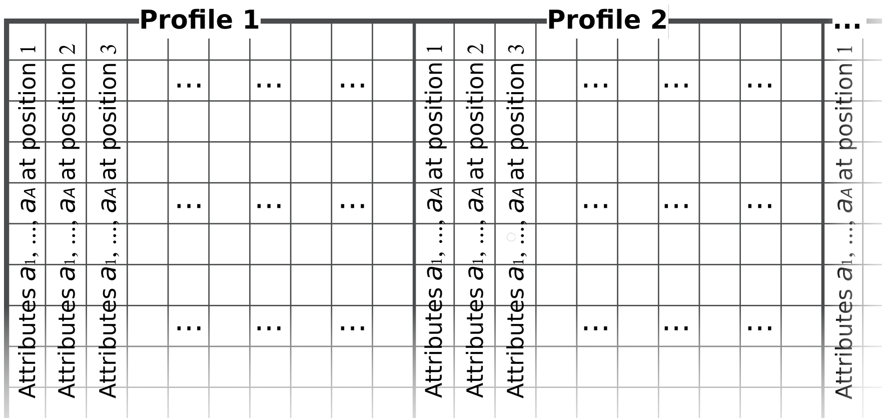

Figure S2. **Profile data organization in memory.**

Such a data structure ensures coalesced and aligned memory access for each profile attribute and maximizes memory throughput.

S1.3 Computation of scores

Scores between query profiles and database profiles are calculated by exploiting massively parallel computing. Thread blocks consisting of 1024 threads process a batch score matrix in parallel (Figure S3), where the threads of each block also run in parallel. A block dimension of 32x32 allows for highly efficient memory accesses, as the calculation of 1024 cells of the score matrix requires reading only 32 query and 32 database A-dimensional profile vectors (attribute values at a certain position).

The threads of a block first cache $A \times (32 + 32)$ profile data values, calculate the scores for the locations corresponding to the query and database profile positions being processed, and write the 1024 calculated scores to memory. To achieve maximum memory throughput, the threads coalesce reads and writes using shared memory (cache).

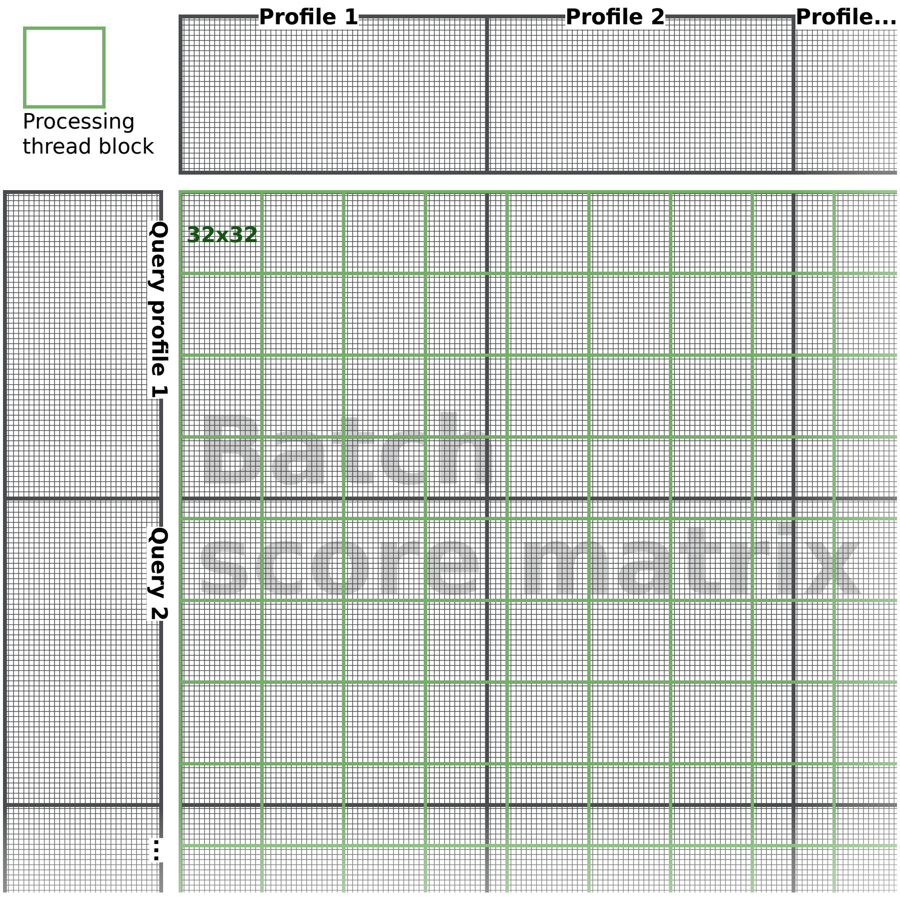

Figure S3. **Illustration of score matrix computation.** Green squares represent 32x32 thread blocks running in parallel. Each thread block calculates 1024 scores in parallel for a small part of the batch score matrix. The smallest cells in the batch score matrix represent single matrix cells.

S1.4 Computation of DP matrices

The computation of DP matrices is much more complicated. Occupancy of running threads is limited by data dependencies: each DP matrix cell depends on its upper, left, and diagonal neighbors. Therefore, the calculation of a cell in the DP matrix requires the values of the whole submatrix, excluding the cell itself at the bottom-right corner, to be known.

The solution for keeping the thread occupancy high is to arrange the DP matrix in diagonal blocks and process these blocks iteratively in parallel (Figure S4). Similarly to the approach discussed in Section S1.3, two-level parallelism emerges.

A block of 32 threads processes a diagonal block of 1024 matrix cells in parallel, where the processing also includes updating backtracking information. Concurrently, multiple thread blocks process multiple diagonal cell blocks at the same time. Due to data dependencies, the processing, however, is arranged in iterations.

Dependencies between data imply that only the diagonal cell blocks along one anti-diagonal (highlighted in Figure S4) of a DP matrix, corresponding to one pair of a query and a database

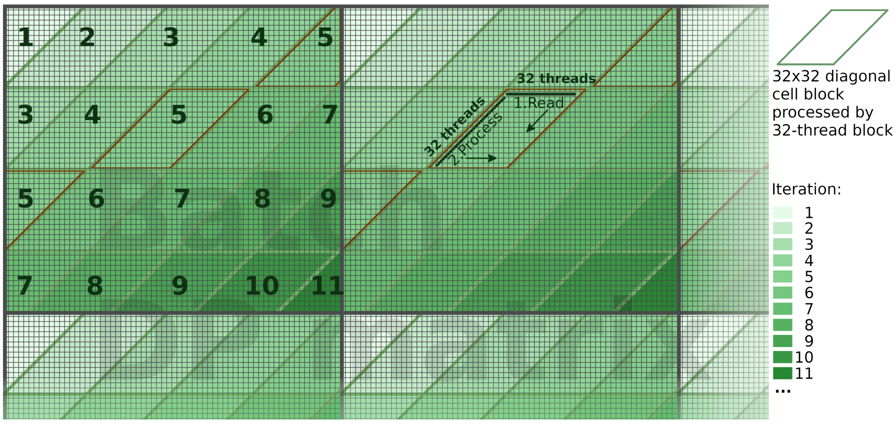

Figure S4. **Illustration of DP matrix computation.** DP matrices corresponding to different pairs of query (along the y-axis) and database profiles (along the x-axis) are delineated by thick grey lines. Multiple DP matrices are processed at the same time. The processing is arranged in iterations. At each iteration, 32-thread blocks process one anti-diagonal of diagonal cell blocks from each DP matrix in parallel. Diagonal cell blocks processed in the same iteration are numbered with the same iteration number. For illustration, diagonal cell blocks of iteration 5 are highlighted in orange. The smallest cells in each diagonal cell block represent single cells of DP matrices.

profile, can be processed at a time. Yet, multiple anti-diagonals from multiple DP matrices can be processed in parallel. Accordingly, thread blocks iteratively process the whole batch DP matrix, i.e., multiple DP matrices, at the same time. High occupancy is achieved by processing at each iteration anti-diagonals from all DP matrices being calculated.

The processing of anti-diagonals at each iteration requires only a small fraction of the calculated values of the diagonal blocks from the previous iteration. These values correspond to the bottom row and the right-most column of each diagonal cell block. Hence, the memory requirements to calculate the whole batch DP matrix are $O(\sum_i l_i)$, where l_i is the length of database profile i, and the sum runs over all profiles in the current chunk of the profile database.

The maximum memory throughput for this configuration of computation is achieved by coalesced reads and writes. The 32 threads of each thread block read and write the data of the corresponding diagonal cell block (including the scores described in Section S1.3) in parallel using shared memory. Instruction throughput is maximized by that the 32 threads of each thread block process all 32 rows of the corresponding diagonal cell block independently (but synchronously) and in parallel (Figure S4).

S1.5 Impact on sensitivity and alignment quality

COMER2 introduced an entirely different software architecture compared to the previous version. These differences include changes in data types, new data structures, parallel algorithms, and instruction set architecture. They alone are sufficient to affect sensitivity and final alignments (and hence their quality). For example, 7 significant digits of the 32-bit floating-point number, the main data type used in COMER2, imply rapid error growth when performing millions of fast but less accurate mathematical operations and functions on these numbers.

The result of the calculation of batch matrices was mostly affected upon the introduction

of Schraudolph's approximation to exponential and logarithmic functions (Schraudolph, 1999). They take only one instruction (multiply-add) but cause relative errors of up to 6%. To counterbalance the accumulation of errors, we eliminated the down-rounding of the similarity score between profile context vectors (Margelevičius, 2018), which had been used to produce slightly shorter local alignments. These changes led to an increase in sensitivity score on the validation dataset (Section S2.1.1) from $\mathrm{ROC}^{\mathrm{S}}_{4\mathrm{K}} = 0.8250$ to 0.8360, where $\mathrm{ROC}^{\mathrm{S}}_{4\mathrm{K}}$ is the normalized area under the ROC curve up to 4000 false positives (Section S2.4).

The extent of local alignments produced by COMER is controlled by the posterior probability threshold option MINPP in the options file `options.txt` (please see the README file in the software package). To take into account the impact of the discussed changes on resulting alignments, the default value of this option was adjusted based on ROC analysis of high-quality alignments on the validation dataset in the local and global evaluation modes (Section S2.5). A value of 0.28 was found to be the best compromise (Table S1) and was set as the default value. Although it does not differ much from the default value used in the previous version (0.30), the user may wish to experiment with different values of the option MINPP.

MINPP	$\mathrm{ROC}^{\mathrm{LQ}}_{200}$	$\mathrm{ROC}^{\mathrm{GQ}}_{1\mathrm{K}}$
0.24	0.8298	0.6285
0.26	0.8482	0.6084
**0.28**	**0.8722**	**0.5875**
0.30	0.8763	0.5665
0.32	0.8816	0.5433
0.34	0.8837	0.5197

Table S1. **Impact of the option MINPP on alignment quality.** $\mathrm{ROC}^{\mathrm{LQ}}_{200}$ is the normalized area under the ROC curve up to 200 low-quality alignments (LQAs) and evaluates alignments in the local mode. $\mathrm{ROC}^{\mathrm{GQ}}_{1\mathrm{K}}$ is the normalized area under the ROC curve up to 1000 LQAs and evaluates alignments in the global mode. Bold values represent the best trade-off between $\mathrm{ROC}^{\mathrm{LQ}}_{200}$ and $\mathrm{ROC}^{\mathrm{GQ}}_{1\mathrm{K}}$. $\mathrm{ROC}^{\mathrm{LQ}}_{200} = 0.8730$ and $\mathrm{ROC}^{\mathrm{GQ}}_{1\mathrm{K}} = 0.5674$ for COMER v1.5 using its default value of 0.30 for MINPP.

S2 Evaluation

S2.1 Datasets

S2.1.1 SCOPe dataset The SCOPe dataset was compiled from SCOPe database v2.03 (Fox *et al.*, 2013) of protein domains filtered to 20% sequence identity. It included 4900 domains from 547 folds (Margelevičius, 2018). Out of these domains, 1722 representing each superfamily, but including every fourth domain of larger superfamilies, were used as queries.

1112 domains from the remaining folds were used as the validation dataset to adjust the default value for the user-configurable option MINPP (Section S1.5).

Two categories of multiple sequence alignments (MSAs) were used to construct profiles for the domain sequences. The first category of MSAs was obtained by running for each sequence PSI-BLAST (Altschul *et al.*, 1997) (v2.2.28+) for six iterations using a sequence inclusion threshold of 10^{-5} and soft masking of low complexity regions against the UniRef50 sequence database (Suzek *et al.*, 2015). The second category of MSAs was obtained by running HHblits (Remmert *et al.*, 2012) for three iterations using default settings against the UniProt20 database of profile HMMs. Only statistically significant matches were included in MSAs (Margelevičius, 2018).

Secondary structure predictions were calculated from the MSAs using PSIPRED (Jones, 1999).

S2.1.2 Simulated dataset The simulated dataset was created by concatenating two sets of profiles. First, one million random profiles were generated using the tools published, and the procedure described previously (Margelevičius, 2019). Following the procedure, profiles of various (effective) numbers of observations (sequences) and lengths were generated by adding random noise ($r = 0.03$) to 1012 (seed) profiles representing unrelated UniRef50 sequences, randomly sampling fragments of length $s = 9$, and splicing the fragments together.

The initial effective number of observations (ENO) as a parameter for generating profiles was sampled from a normal distribution with a mean of 6 and a standard deviation of 1.5 and then floored to the nearest multiple of 2 with a minimum of 2 and a maximum of 12. The final ENO was not strictly controlled, and profiles were allowed to take on different values in the vicinity of sampled initial values.

The profile length was sampled from a gamma distribution with a shape of 2 and a scale of 125 and then rounded to the nearest integer. The minimum profile length was set to 20. This setting implies a mean profile length of approximately 250. Figure S5 shows the distribution of the number of generated profiles.

The random MSAs from which the profiles were constructed are available at
https://sourceforge.net/projects/comer2/files/comer2-pub-data-2.02

The other part of the simulated dataset was 1284 profiles constructed for the sequences of randomly selected PDB (Berman *et al.*, 2000) structures sharing less than 20% sequence identity at the domain level. The COMER profiles were constructed from MSAs obtained from HHpred PDB70 database 190918 (Zimmermann *et al.*, 2018). The MSAs were converted from a3m to FASTA format using the `reformat.pl` program from the HH-suite3 software package (Steinegger *et al.*, 2019). The HHsearch/HHblits profiles were used directly from HHpred PDB70 database 190918.

The simulated dataset contained 1 001 284 profiles in total. The size of the COMER profile database created for this dataset was 53GB. The corresponding size of the HHsearch/HHblits profile database was 29GB. Hence, COMER2 was required to read nearly twice as much data for its searches.

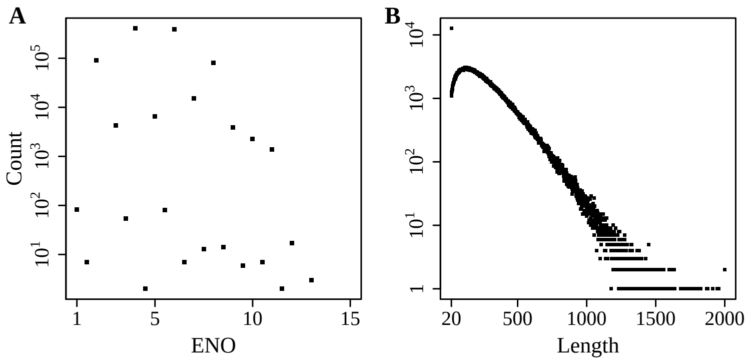

Figure S5. **Distribution of the number of random profiles by (A) ENO and (B) length.**

Query profiles for searching the simulated dataset were also obtained from HHpred PDB70 database 190918 entries chosen at random. There were 128 query profiles.

S2.2 Command-line options of profile-profile alignment methods

The latest versions of the following profile-profile alignment methods were evaluated: COMER versions v2.2.1 and v1.5.1, HHsearch and HHblits v3.2.0 (Steinegger *et al.*, 2019), FFAS (Jaroszewski *et al.*, 2011), and COMPASS v3.1 (Sadreyev and Grishin, 2008).

COMER search can be configured using the options file `options.txt`. The tests were performed with default values set for the options, including an E-value threshold of 10 (`EVAL=10`). The exceptions were `NOHITS=7000` and `NOALNS=7000` giving the maximum number of hits to display (never reached), and `SSEMODEL=1` specifying the use of statistical model 1 for the SCOPe dataset when producing local alignments (default `MINPP` value). The number of GPUs to be used and GPU memory limits were specified using the command-line options `--dev-N` and `--dev-mem`, respectively. The expected proportion of significant alignments was specified by setting `--dev-pass2memp=40` and `--dev-pass2memp=1` for the SCOPe and the simulated dataset, respectively.

HHsearch and HHblits searches were configured similarly:
`-mact` m `-e 10 -p 30 -E 10 -Z 7000 -z 1 -B 7000 -b 1 -v 0 -alt 1 -cpu` c
where $m = 0$ when producing maximally extended alignments and $m = 0.3$ otherwise, which yielded better alignment quality than a default value of 0.35 without affecting sensitivity. c is the number of CPU cores. HHblits was additionally configured to run one iteration: `-n 1`.

Default values for options were used for FFAS. COMPASS was configured with the options `-e 10 -v 3000` specifying an E-value threshold of 10 and the (allowed) maximum number of hits in output.

Profiles for each of these methods were constructed using default settings. Secondary structure predictions were incorporated into COMER and HHsearch/HHblits profiles using the `makepro.sh` and `addss.pl` programs from the respective software packages. HHsearch/HHblits profiles from HHpred PDB70 database 190918 contained DSSP secondary structure assignment (Kabsch and Sander, 1983) and/or PSIPRED predictions and were used as-is.

S2.3 Running time

The running time of profile-profile alignment methods was measured by the Linux `time` command. The mean and standard deviation were estimated by repeating the measurement three times.

S2.4 Sensitivity

Sensitivity was measured by the number of identified true positives (TPs). A pair of aligned domains that belonged to the same SCOPe superfamily or shared statistically significant structural similarity (DALI (Holm *et al.*, 2008) Z-score ≥ 2) was considered a correct match (TP). For the simulated dataset, the analysis was performed at the domain level.

Aligned pairs that did not meet the above criteria but belonged to the same SCOPe fold were considered to have an unknown relationship and were ignored. Other aligned pairs or alignments involving a random profile were false positives (FPs).

S2.5 Alignment quality

Alignment quality for the SCOPe dataset was evaluated using a reference-free approach (Margelevičius and Venclovas, 2010; Margelevičius, 2016, 2018).

An alignment between two domains was considered to be of high quality if the most accurate structural model generated for one of the two domains, using the other domain as a template, was similar to the real structure. Structural models were generated using MODELLER v9.15 (Šali and Blundell, 1993). TM-score (Zhang and Skolnick, 2004) was used to evaluate the structural similarity between a model and the native structure. An alignment was considered a high-quality alignment (HQA) if it caused a statistically significant similarity, TM-score ≥ 0.4. A low-quality alignment (LQA) was an alignment for which TM-score < 0.2.

Alignments were evaluated in the local and global modes. The global mode was used to evaluate the alignment with respect to the whole protein domain, whereas the local mode along the alignment extent.

S3 Results

S3.1 Additional results for the SCOPe dataset

This section presents additional results for the SCOPe dataset.

Figure S6 supplements the first part of Figure 1 in the main text. Figure S6D and H show alignment quality evaluated in the global mode when COMER, HHsearch, and HHblits were configured to produce local alignments. Alignment quality evaluated in the local mode is shown in Figure S6E and I. Here, COMER, HHsearch, and HHblits were configured to extend alignments maximally. The results of COMER v2.2 in Figure S6 show consistent improvement in alignment quality.

The quality of alignments was also measured by counting the number of HQAs that resulted in structural models with a TM-score ≥ 0.5. A TM-score ≥ 0.5 usually indicates a close homology between proteins identified by homology search and is an indicator of a high-quality structural model sharing the same fold with a template structure. This threshold is used for evaluating full-length protein target models.

Figure S7 shows that using this threshold, COMER v2.2 produces a greater number of HQAs than the other tested methods irrespective of the evaluation mode and the category of alignments (local or maximally extended).

The running time of COMER v2.2, HHsearch v3.2, and HHblits v3.2 is shown in Figure S8. Similarly to the results observed for the simulated dataset, COMER v2.2 achieves near-perfect scaling even on the relatively small SCOPe dataset. And its running time weakly depends on the GPU memory used.

The results of the running time of HHsearch v3.2 shows that distributing work across a larger number of (physical) cores is not very efficient for a relatively small dataset.

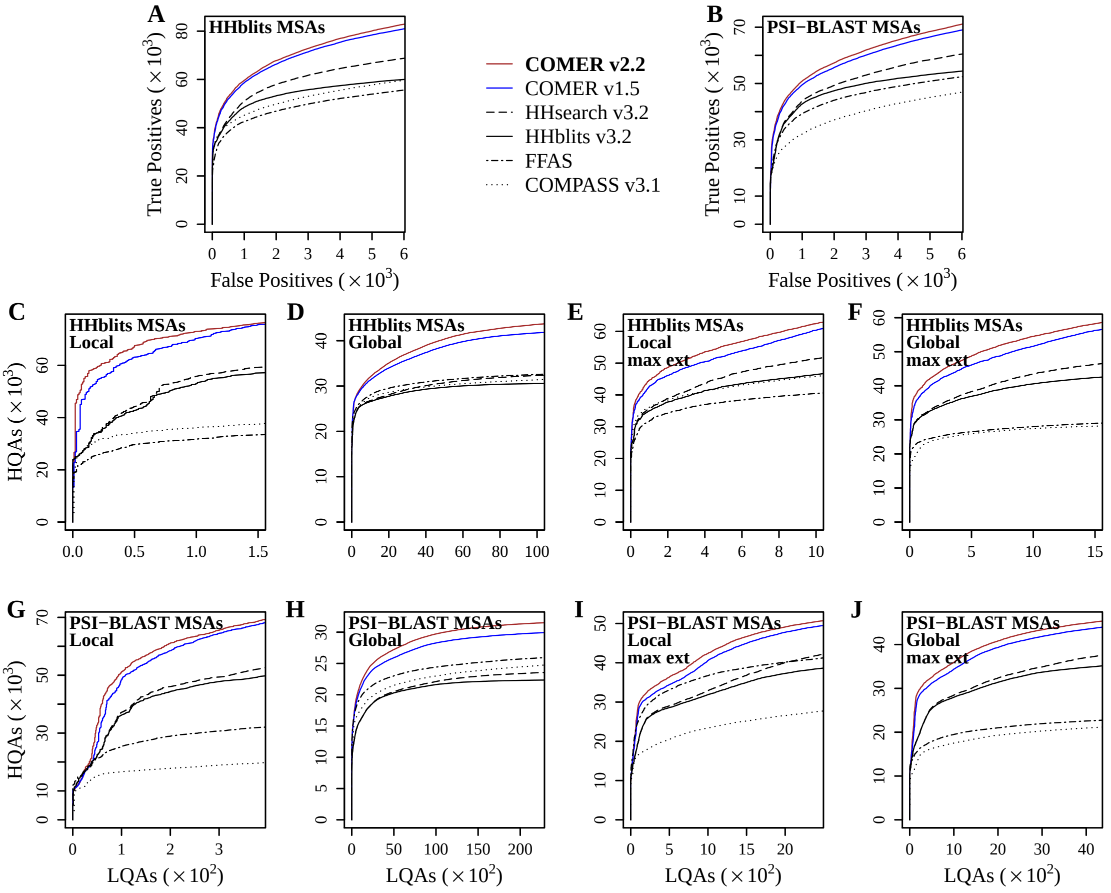

Figure S6. **Results for the SCOPe dataset.** (A,B) Sensitivity. (C–J) Alignment quality evaluated in (C,E,G,I) the local and (D,F,H,J) the global evaluation mode. (E,F,I,J) Maximally extended (max ext) COMER, HHsearch, and HHblits alignments. Profiles were constructed from (A,C–F) HHblits and (B,G–J) PSI-BLAST MSAs.

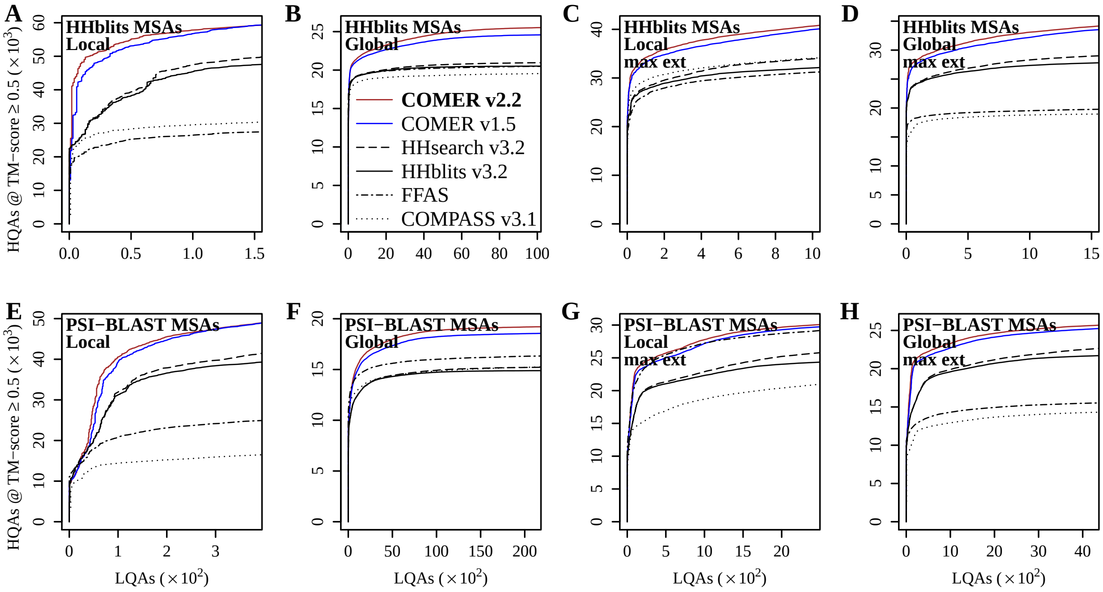

Figure S7. **Alignment quality for the SCOPe dataset.** Alignment quality is measured by the number of HQAs leading to structural models with a TM-score $\geq$ 0.5. (A,C,F,G) Alignment quality evaluated in the local mode. (B,D,F,H) Alignment quality evaluated in the global mode. (C,D,G,H) Alignment quality of maximally extended (max ext) COMER, HHsearch, and HHblits alignments. (A–D) Results for profiles constructed from MSAs obtained using HHblits. (E–H) Results for profiles constructed from MSAs obtained using PSI-BLAST.

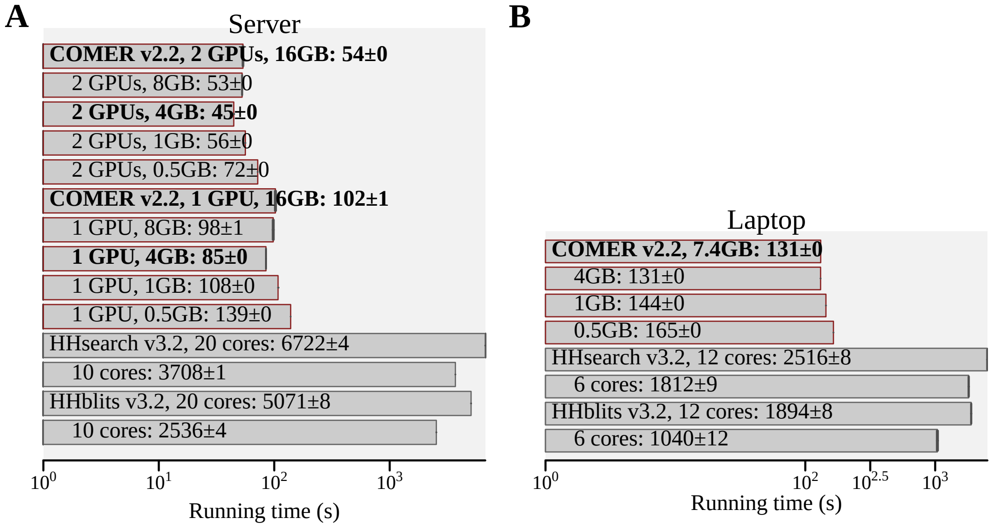

Figure S8. **Running time for the SCOPe dataset on (A) the server and (B) the laptop.**

S3.2 Running time for the simulated dataset

The running time of COMER v2.2, HHsearch v3.2, and HHblits v3.2 for the simulated dataset is shown in Figure S9. Figure S9 supplements the second part of Figure 1 of the main text and shows running times for different settings.

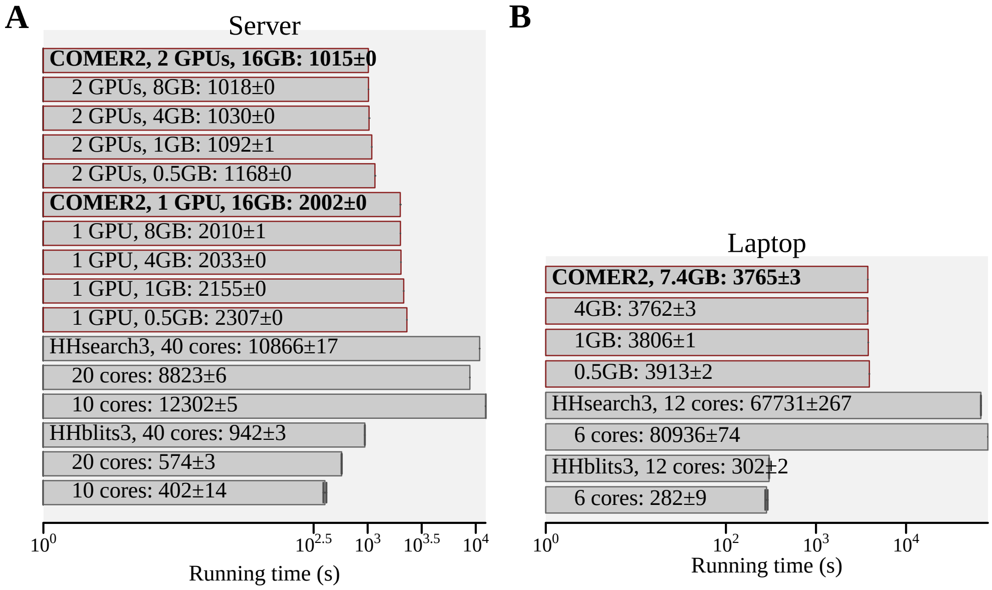

Figure S9. **Running time for the simulated dataset on (A) the server and (B) the laptop.**

S3.3 Benchmark tests on multi-domain proteins

Practical applications involve searching for protein chain sequences. The simulated dataset, therefore, included profiles constructed for full-chain sequences from the PDB. This section reports the results of additional tests performed on multi-domain proteins.

First, profiles and their database were constructed for all 190 multi-domain protein chain sequences of class e from SCOPe v2.03 filtered to 20% sequence identity. Then, all of the profiles were searched against their database.

Figure S10 shows the sensitivity and alignment quality results for the multi-domain proteins. The results of the alignment quality measured by the number of HQAs with a TM-score ≥ 0.5 are shown in Figure S11.

The results show that overall, the COMER method retains the advantage, but the quality of COMER v2.2 alignments evaluated in the local mode (Figure S10C,G and Figure S11A,E) is inferior to that of COMER v1.5. On the other hand, the opposite is observed in the global evaluation mode (Figure S10D,H and Figure S11B,F). Longer alignments produced by COMER v2.2 for TPs (Figure S10A,B) lead to a greater number of HQAs in the global mode at the expense of a lower number in the local mode. Closer inspection also revealed that the differences between COMER v2.2 and v1.5 in the local mode manifest for statistically insignificant alignments.

The running time analysis (Figure S12) shows the efficiency of COMER v2.2. A speedup of greater than a factor of 3000 compared to COMER v1.5 is observed.

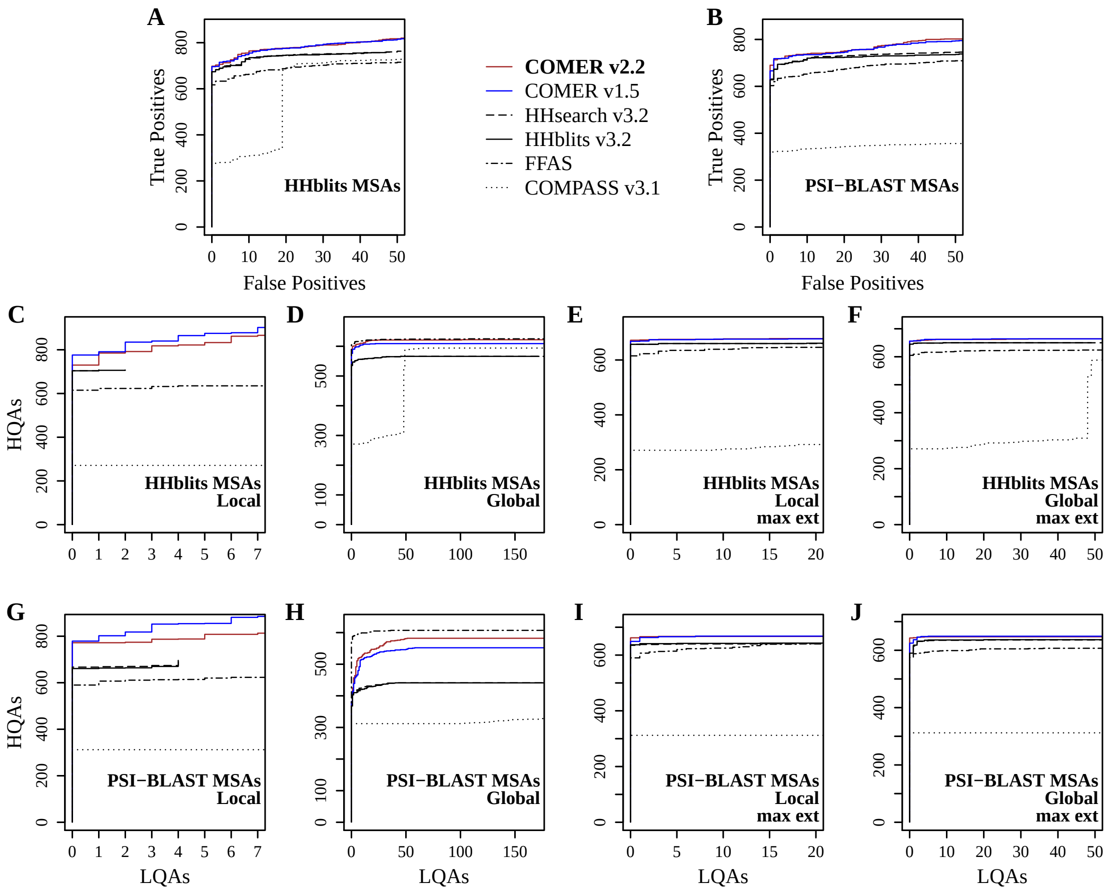

Figure S10. **Results obtained on** 190 **multi-domain proteins.** (A,B) Sensitivity. (C–J) Alignment quality evaluated in (C,E,G,I) the local and (D,F,H,J) the global evaluation mode. (E,F,I,J) Maximally extended (max ext) COMER, HHsearch, and HHblits alignments. Profiles were constructed from (A,C–F) HHblits and (B,G–J) PSI-BLAST MSAs.

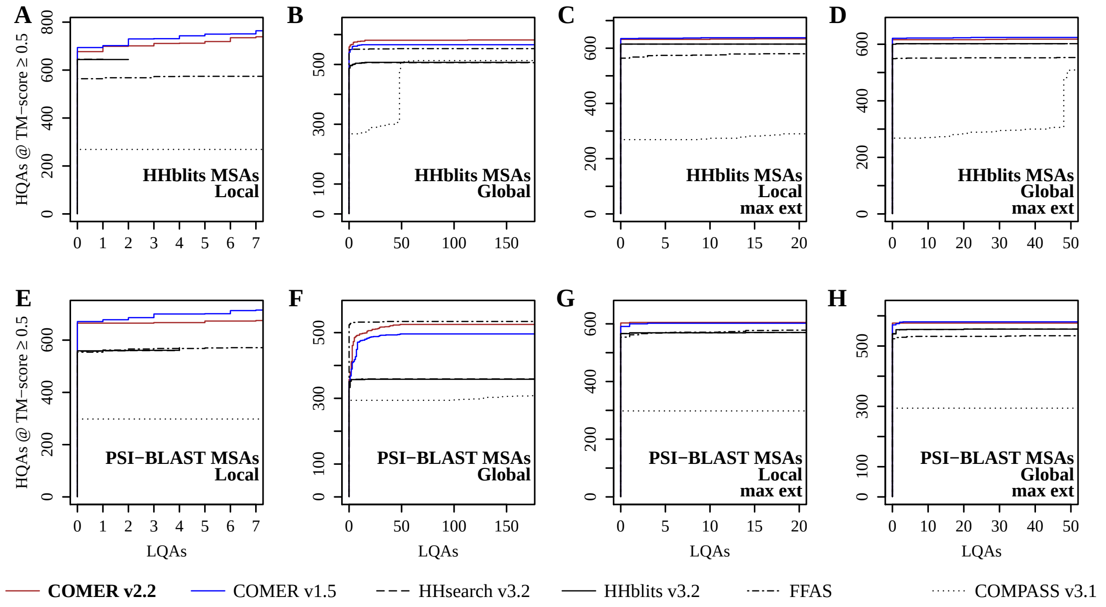

Figure S11. **Alignment quality results on 190 multi-domain proteins.** Alignment quality is measured by the number of HQAs leading to structural models with a TM-score ≥ 0.5. (A,C,F,G) Alignment quality evaluated in the local mode. (B,D,F,H) Alignment quality evaluated in the global mode. (C,D,G,H) Alignment quality of maximally extended (max ext) COMER, HHsearch, and HHblits alignments. (A–D) Results for profiles constructed from MSAs obtained using HHblits. (E–H) Results for profiles constructed from MSAs obtained using PSI-BLAST.

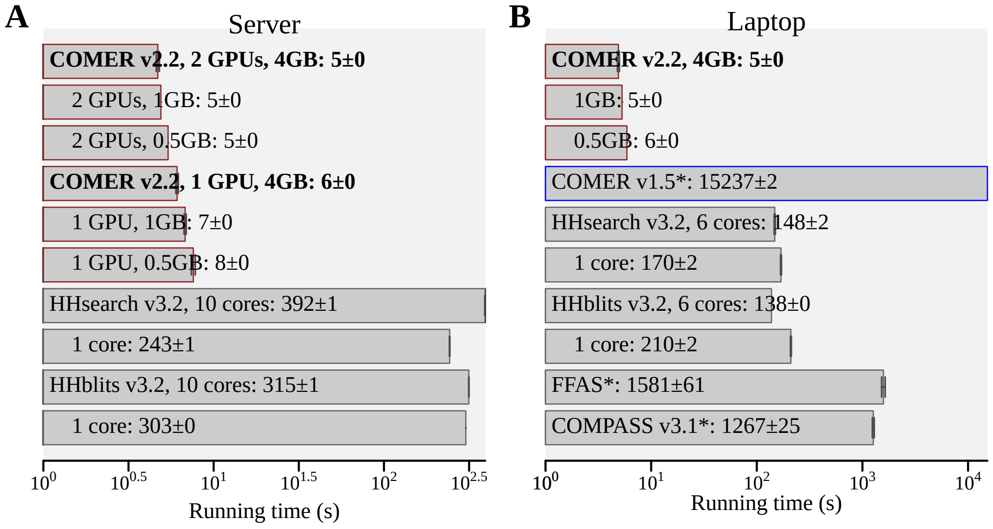

Figure S12. **Running time for the test on 190 multi-domain proteins on (A) the server and (B) the laptop.** *Measured on a cluster with Intel Xeon CPUs E5-2670 v3.

S3.4 Running time of profile and database construction

Fast computation is particularly important for homology search applications. Importantly, data preparation for homology search based on profile-profile comparison and alignment includes the construction of profiles and profile database creation. Figure S13 shows the dependence of the running time of profile construction on the size of the MSAs of native sequences obtained using HHblits (Section S2.1.1). It also shows the total running time required for profile construction and the running time of database construction for the SCOPe dataset. The running times of database construction for the simulated dataset are shown in Figure S14.

These results show that database construction, including the construction of profiles, can be a computationally demanding procedure. Approaches for accelerating profile construction, therefore, would greatly facilitate the data preparation phase. Parallel algorithms for processing multiple MSAs on the GPU will be considered in future work.

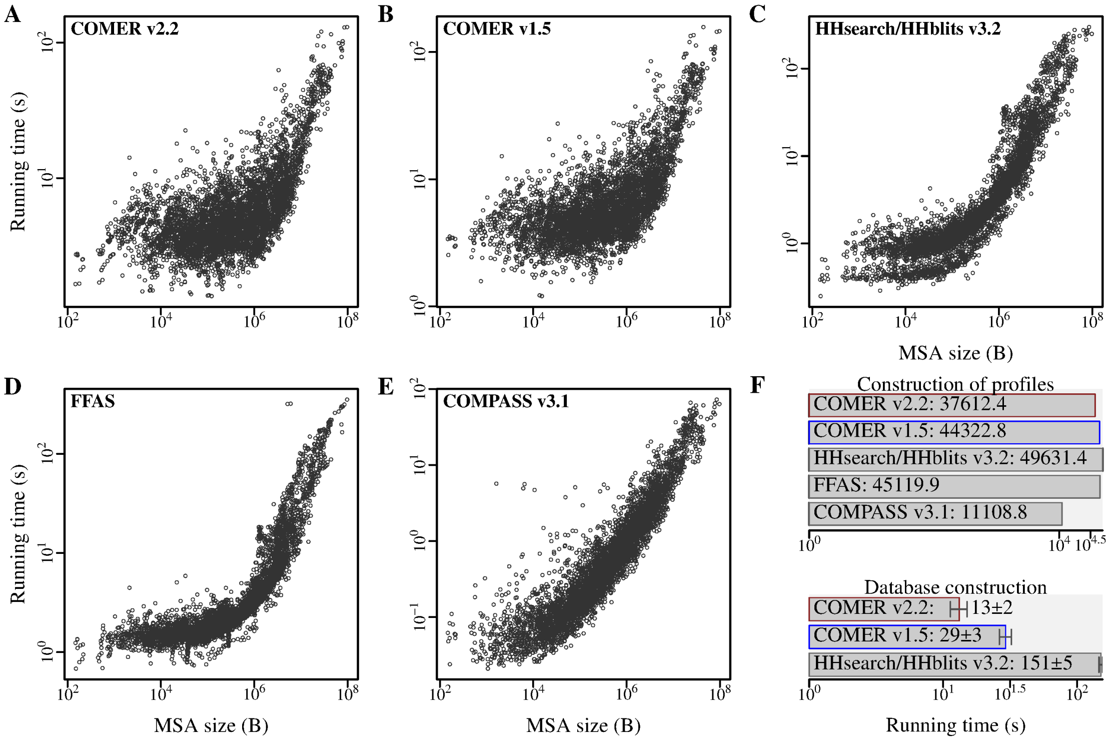

Figure S13. **Running time of the construction of profiles and databases for the SCOPe dataset.** (A–E) Running time of profile construction plotted against the size of MSAs in FASTA format for each method. Profile construction for COMER (both versions) and HHsearch/HHblits includes predicting secondary structure and its incorporation into the profile. (F) Total running time of profile construction over all of the 4900 domains (top) and running time of database construction (bottom). Profile databases for FFAS and COMPASS correspond to concatenated profile files, and the running time of their construction is not shown. The running time for COMER v2.2 is that of `makedb` + `db2bin`. 16 CPU cores were used to construct the HHsearch/HHblits profile database. Measurements were performed on a cluster with Intel Xeon CPUs E5-2670 v3.

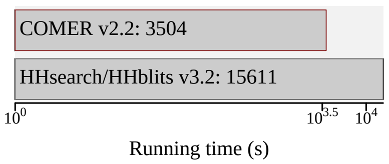

Figure S14. **Running time of database construction for the simulated dataset on the Server using 20 CPU cores.** The running time for COMER v2.2 is that of `makedb` + `db2bin`. Measurements were performed once.

S4 Software and data availability

The COMER v2.2 software is available at

- https://sourceforge.net/projects/comer2 and
- https://github.com/minmarg/comer2

The software package includes stand-alone installers for Windows and Linux.

Dataset information, alignment data, scripts, and one million random MSAs used to construct profiles for the simulated dataset are available at

- https://sourceforge.net/projects/comer2/files/comer2-pub-data-2.02

COMER2 profile databases for homology searches are available at

- https://sourceforge.net/projects/comer2/files/comer2-profile-databases-2.02

Currently, profile databases of PDB70 and SCOP70 v1.75 entries are available.

References

Altschul, S. F., Madden, T. L., Schäffer, A. A., Zhang, J., Zhang, Z., Miller, W., and Lipman, D. J. (1997). Gapped BLAST and PSI-BLAST: a new generation of protein database search programs. *Nucleic Acids Res*, **25**(17), 3389–3402.

Berman, H. *et al.* (2000). The Protein Data Bank. *Nucleic Acids Res*, **28**, 235–242.

Durbin, R., Eddy, S. R., Krogh, A., and Mitchison, G. (1998). *Biological Sequence Analysis: Probabilistic Models of Proteins and Nucleic Acids*. Cambridge University Press.

Fox, N., Brenner, S., and Chandonia, J. (2013). SCOPe: Structural classification of proteins–extended, integrating SCOP and ASTRAL data and classification of new structures. *Nucleic Acids Res*, **42**(D1), D304–D309.

Holm, L., Kääriäinen, S., Rosenström, P., and Schenkel, A. (2008). Searching protein structure databases with DaliLite v.3. *Bioinformatics*, **24**(23), 2780–2781.

Jaroszewski, L., Li, Z., Cai, X. H., Weber, C., and Godzik, A. (2011). FFAS server: novel features and applications. *Nucleic Acids Res*, **39**, W38–W44.

Jones, D. T. (1999). Protein secondary structure prediction based on position-specific scoring matrices. *J Mol Biol*, **292**(2), 195–202.

Kabsch, W. and Sander, C. (1983). Dictionary of protein secondary structure: pattern recognition of hydrogen-bonded and geometrical features. *Biopolymers*, **22**(12), 2577–2637.

Margelevičius, M. (2016). Bayesian nonparametrics in protein remote homology search. *Bioinformatics*, **32**(18), 2744–2752.

Margelevičius, M. (2018). A low-complexity add-on score for protein remote homology search with COMER. *Bioinformatics*, **34**(12), 2037–2045.

Margelevičius, M. (2019). Estimating statistical significance of local protein profile-profile alignments. *BMC Bioinformatics*, **20**, 419.

Margelevičius, M. and Venclovas, Č. (2010). Detection of distant evolutionary relationships between protein families using theory of sequence profile-profile comparison. *BMC Bioinformatics*, **11**, 89.

Remmert, M., Biegert, A., Hauser, A., and Söding, J. (2012). HHblits: lightning-fast iterative protein sequence searching by HMM-HMM alignment. *Nat Methods*, **9**(2), 173–175.

Sadreyev, R. and Grishin, N. (2008). Accurate statistical model of comparison between multiple sequence alignments. *Nucleic Acids Res*, **36**(7), 2240–2248.

Schraudolph, N. (1999). A fast, compact approximation of the exponential function. *Neural Comput*, **11**(4), 853–862.

Steinegger, M., Meier, M., Mirdita, M., Vöhringer, H., Haunsberger, S., and Söding, J. (2019). HH-suite3 for fast remote homology detection and deep protein annotation. *BMC Bioinformatics*, **20**, 473.

Suzek, B., Wang, Y., Huang, H., McGarvey, P., Wu, C., and the UniProt Consortium (2015). UniRef clusters: a comprehensive and scalable alternative for improving sequence similarity searches. *Bioinformatics*, **31**(6), 926–932.

Šali, A. and Blundell, T. L. (1993). Comparative protein modelling by satisfaction of spatial restraints. *J Mol Biol*, **234**(3), 779–815.

Zhang, Y. and Skolnick, J. (2004). Scoring function for automated assessment of protein structure template quality. *Proteins*, **57**(4), 702–710.

Zimmermann, L. *et al.* (2018). A completely reimplemented MPI bioinformatics toolkit with a new HHpred server at its core. *J Mol Biol*, **430**, 2237–2243.
